# Introductory Lecture, before the Class of the Baltimore College of Dental Surgery

**Published:** 1846-12

**Authors:** A. Westcott

**Affiliations:** Professor of Operative and Mechanical Dentistry; furnished for publication by request of the Class.


### ARTICLE IV.

*Introductory Lecture, delivered before the Class of the Baltimore College of Dental Surgery, at the session of* 1846–47. By A. Westcott, M. D., D. D. S., Professor of Operative and Mechanical Dentistry; furnished for publication by request of the Class.

*Gentlemen—Students of the*
*Baltimore College of Dental Surgery:*

You have doubtless been attracted hither by the belief that you could here enjoy pre-eminent advantages for qualifying yourselves for the profession of your choice; a profession alike useful and honorable.

Believing that the ardor and success with which you will pursue your studies, will in some measure correspond with your conviction, that you are here on the high road which will conduct you to the object of your ambition, I propose, in entering upon the duties to which I have been invited, to call your attention to the question: *do dental colleges possess peculiar advantages over any other means of securing a dental education?*

This, gentlemen, I conceive to be a question, not only of interest to you personally, but one involving, in a high degree, the future usefulness and respectability of our profession. As we cannot refer for proof to actual results in institutions of long standing, it becomes necessary to look at the distinctive features of the profession, and institute an examination into its nature, its attributes and its requisitions.

We shall find this, in itself considered, by no means a barren or unprofitable field of investigation. Nothing can, indeed, be of greater importance to the student in any profession, than to learn early what branches of knowledge or science can essentially contribute to his success in his specific calling. Every science which is connected with matter, is necessarily connected with other sciences; and, in most instances, it very materially depends upon many others for its perfection. It would, for example, be quite impossible to conceive of natural philosophy, in the present state of that science, without the aids it has derived from chemistry; and chemistry, in turn, is equally indebted to this, her sister science. A similar remark will apply to every science connected with material objects, however distinct either may be regarded or taught. No very great proficiency can be made in any one, without some knowledge of all the others.

The science of *medicine*, which has for its object the prevention and cure of disease, is one of vast magnitude. If it does not include, it levies frequent and extensive contributions on every other science. When viewed with reference either to its comprehensiveness or importance, it may emphatically be regarded the primary, in the whole system of sciences.

But owing to the extent of the field which the general science of medicine embraced, divisions and sub-divisions, for convenience sake, have from time to time been made.

The necessity for such division was enhanced, as the field was explored and cultivated, by bringing to its aid the collateral sciences. Nor hardly need it be observed, that the progress towards perfection, which was made in the several departments, kept pace with the attention which each branch specifically received. Nothing can be more striking than the change which has been wrought in general surgery, since it has become a distinct field of investigation and practice.

True, the common physician frequently performs many of the minor operations, with a good degree of skill. But of whom did he obtain this skill? Was it from the physician, who had only pursued surgery, in common with the other branches composing the general science of medicine? By no means. No, he was taught by a master, who had become such by devoting his special attention to this particular subject, perhaps for a long series of years.

This division, then, has wrought a two-fold good. It, on the one hand, has perfected a specific branch of medicine; and, on the other, has reflected back this perfection upon the general practitioner; imparting to him a skill before unknown. Many other divisions have been made, creating distinct departments of the healing art, and the result has been equally marked and happy.

The oculist has given eyes to the blind; the aurist has caused the deaf to hear, and the *dental surgeon*, with science no less profound, has relieved suffering humanity from the most excruciating pains that "flesh is heir to;" and, by preserving or restoring the organs of mastication, has imparted youthfulness to age, restored to health the enervated dyspeptic; for ugliness has substituted symmetry of features, and has restored distinct enunciation for the broken, inarticulate lisping of toothless decrepitude.

Although these several divisions are spoken of as constituting so many distinct branches of the healing art, yet it is by no means to be understood that the student in either can be excused, hence, from acquainting himself with the general subject of them all—the human system. Neither does the fact that these several divisions depend upon the same general principles, interfere with their being both studied, and taught as distinct branches of science. The wholesale dealer, who sells but a single class of articles, would by no means relinquish his right to be ranked as a merchant, because his neighbor, a retailer, sold every kind of merchandise from a single counter.

Nor is it hard to perceive that the latter, with a given outlay of capital, could much more perfectly manage his single branch of business. Professional men too often become retailers in science. In other words, they frequently divide their time and intellectual capital among several branches, any one of which, to be well managed, should receive the whole.

Now the divisions alluded to in medical science, are for convenience sake alone; and, but for this consideration, might be regarded arbitrary. Could any institution be sufficiently extensive to give adequate attention to every department, there would be no objection to the same institution embracing the whole; and could the student devote the adequate time and means, and did he possess the mental capacity successfully to cultivate so extensive and varied a field of pursuit, there could certainly be no objection to his assuming the task. Should he be successful, he would have the satisfaction of having accomplished the work of many. Such prodigies, however, have hitherto been rare. If we consult the history of the arts and sciences, and especially that of our own day, a time when such prodigious advances are made in both, we shall not fail to recognize the *labor-dividing system*, as the most prominent and efficient agent in giving such impetus to the march of improvement, in every department of labor and learning.

Permit me now to call your attention to that department of the healing art in which you are particularly interested, and which it is your purpose here to cultivate—dental surgery, its attributes, and the requisite qualifications in order to become a skilful practitioner in this single department.

I doubt not that if I am successful in presenting these attributes, and the requisitions which this subject, if properly mastered, will make upon your time and attention, two conclusions will force themselves upon you, viz. 1st. That it will furnish a most ample field for all your time and talents; that if you cultivate your profession efficiently, you will never have occasion, as did Alexander, to weep because there is nothing left to conquer.

2d. That institutions, specifically devoted to dental surgery, are necessary, in order to the full development of the principles it includes.

I have already observed that the divisions in medical science are made for convenience sake, and it will be proper here to add, that the *extent* of the field, embraced in the different departments, is fixed on the same principle.

What is the field allotted to the dental surgeon?

The term dental surgery does not, if construed literally, convey an adequate idea of what is, by common consent, included under it. Although this term is descriptive of the chief business of the dentist, and gives his department "a local habitation and a name," yet it by no means embraces the entire field of his inquiries, or even his operations. While it is the business of the dental surgeon to inquire into and treat the disease of a specific class of organs, it is also no less his duty to ascertain, if possible, the *cause* of such disease, its connection with other parts, and whether his remedies are to be applied directly, or whether they are not to be directed to the overcoming of some latent difficulty, antecedent to the most prominent disease. In other words, his province and duty is not merely to treat these organs as though they were isolated portions of the system, but as parts of the general system, governed, in many particulars, by the same laws, influencing and being influenced by every other organ.

Hence, his inquiry should be directed to the investigation of every influence which can be supposed to have a bearing upon the diseases of this specific class of organs.

His duty stops not here. It is not only his business to weigh the influence which other organs may exert upon the teeth, but he is also to investigate how far the diseases to which the teeth and mouth are subject, may, in turn, derange the other portions of the system. His field, then, is by no means a contracted one. The dental student is not only to study these particular organs, their immediate connections, their specific diseases, and their peculiarities; but if the view I have taken be a correct one, he should become acquainted with the laws of the entire system, together with those of each organ, their mutual connections and dependencies.

Again, if this view of the subject be correct, no other facts or considerations need be presented, to exhibit most clearly the necessity of a knowledge of general anatomy, which alone can give an adequate idea of the structure and the relative arrangement of the various parts constituting the human frame; even allowing that any organ might be studied, isolated from the rest.

But let us see what progress the dental student would make, in comprehending those parts belonging strictly to the teeth.

For example, on examining the structure of a tooth, he finds it endowed with a nerve, and is disposed to learn its origin and office. He does not pursue his investigation far, before he discovers that it is not an independent nerve, but is simply a branch of another. If he pursues his investigation still farther, he discovers that this last is also a branch of a primary nerve. If he still continue his search, he will find that the original nerve has its origin in the brain; that it gives off first three main branches, and that these give rise to more than twenty other branches or twigs, distributed to different parts, *one* of which supplies the teeth.

He finds, too, that all the remaining branches of this original nerve go to supply other parts of the head, neck and face. Now, on the supposition that he started with the determination of confining his investigation to this *dental* nerve, he will find himself most awkwardly situated—in a labyrinth which he little anticipated.

Should he decide to pursue this investigation, and trace the connection of these different parts, thus established, and study the various sympathies which must necessarily exist between them, whether in a healthy or diseased state, will it be said he has travelled out of his legitimate sphere?

But he is not yet at the end of his difficulties. If he examine more minutely still, he will find this original nerve, either directly or indirectly, connected with every other nerve in the human frame.

If such be the fact, how clear is the futility of attempting to study any part, as isolated from the rest of the system. As well might one attempt to comprehend a complicated piece of machinery, by an examination of a single wheel.

"All are but parts of one stupendous whole," and although every tissue and organ has some peculiarity, by which it is distinguished from all the rest, yet the condition of each is modified by its connections with other parts.

How essential is it then, for the dental, as well as the student in every other department of the healing art, to be able to trace these connections of structure, which he can only do, by a knowledge of GENERAL ANATOMY.

Next in importance to the study of the structure, and relative position of the different organs, is that of their office, or functions. These subjects are embraced under *physiology*, and they constitute a field, most interesting to the dental surgeon.

I have spoken of the structural connections of the different organs of the system, and I may remark that a comprehension of these, is chiefly important, as the basis of a knowledge of their functional relations.

As the anatomist finds it impossible to comprehend any organ singly, in consequence of its connection with others, so the physiologist, finds it equally impracticable, to study disconnectedly, the functions of a single organ.

The functions of the human system, the harmonious action of which constitutes health, have been properly compared to a circle; beginning where we may, we find each part intimately connected with another, essential to the whole; and in whatever direction we may pursue our examination, we ultimately arrive at the starting point. If, for instance, we commence our investigation at the *brain*, we find this dependent upon the heart and lungs, for its supply of arterial blood; if upon the *heart*, this is dependent upon the brain, for nervous energy, and upon the lungs to purify its blood; if upon the *lungs* we find that their action cannot be sustained without both the influence of the nervous system, and the action of the heart, to propel through them the vital fluid, necessary for the support of the whole.

Again each of these is dependent on the *stomach*, for its appropriate nourishment, while the stomach in turn requires their combined agency in order to perform properly its functions.

A similar description applies to every organ of the human system.

But let us examine a little more in detail some of the different organs, and their physiological relations, and in doing this, we can find no more appropriate starting point, than the organs of mastication.

Among these we find the *teeth*, which in their perfect state, are exactly adapted to the comminution of the food, while the muscles of the lips, cheeks, and tongue, act as assistants in keeping the food in the best situation, to be most readily acted upon. That it may be properly moistened, and prepared for the stomach, a suit of glands are provided, to secrete a fluid for the purpose, and the stimulous of the food during mastication excites them to action; and lastly, by the action of the tongue and the muscles of the throat, the food thus prepared is propelled to the stomach.

But the food on reaching the stomach, is by no means prepared to furnish nutriment to the system. To meet this exigency, other organs are brought into play, and it undergoes other processes, till finally the entire nutriment it contained, is carried into, and becomes a part of the blood of the system.

But let us for a moment return and inquire whether the dentist is interested in thus pursuing this investigation.

We see in this description a most beautiful connection of organs and functions, and how each, while in a healthy state, conspires, by assisting the others to accomplish the same ultimate result. But let either of these connected links in the chain be impaired or broken, and what is the result? Imperfection, if not disease, pervades the whole.

Let the different muscles, brought into use during mastication cease to act, and the teeth, however perfect in themselves, would become useless; let the teeth be wanting, or imperfect, and the food could neither be perfectly comminuted, nor insalivated; let, in short, mastication as a whole be imperfect, and digestion must necessarily partake of the imperfection, and the stomach, sooner or later, become impaired.

The process of digestion does not consist simply in separating the nutritive portions of the food, as received into the stomach, but rather in an actual decomposition of this food—manufacturing from a heterogeneous, a homogeneous substance, adapted to the wants of the system.

Hence, the evils of indigestion are not simply negative, by giving rise to a want of nutrition; but by imperfect assimilation, improper substances are carried into the circulation, exerting a morbid influence on every portion of the system. But the stomach is not the only, if it is indeed the chief avenue, through which a diseased denture imparts disease to the general system.

Food is not more essential to the maintenance of health and life than pure air. This must also pass through the mouth before it reaches the lungs.

Now, when you know that at every breath, about eight per cent. of all the air inhaled, is actually absorbed by the blood, and know, too, that in its passage to the lungs, it is necessarily contaminated by a diseased mouth and teeth, often to an extent insupportable to a second person, does it not most forcibly exhibit an important connection between the state of the mouth, and the health of every organ of the system, through the medium of respiration.

Again, it is a principle well settled in medicine, that any constant source of local irritation, must inevitably, sooner or later, radiate its influence throughout the whole nervous system, and in every case where the nerves are constitutionally or otherwise weak, it must finally result in general derangement.

Nor is it necessary that this irritation amount to pain, in order to have the result certain, or sometimes even fatal. I need not add, that no individual even had a diseased denture, without such a source of local irritation.

But I cannot now pursue these illustrations farther, nor is this necessary to make it apparent that physiological science, is necessary to the accomplished dental surgeon.

Anatomy and physiology are both strictly elementary branches of general medical science, and when studied, or taught in their purity, pertain simply to the development of structure, relative position, and the offices of the different organs of the human system in a healthy state.

I will next invite your attention to CHEMISTRY, and to a brief consideration of its applications to dental surgery.

This science, although taught in our medical colleges, as an elementary branch of general medicine, is by no means limited in its applications or its resources.

There is perhaps no single science, which has contributed so much to the perfection of all other sciences, as well as the arts, or which has contributed so much to the melioration of the condition of the human race, as has chemistry.

If the chemist has not succeeded in finding the philosopher's stone, which could *directly* transmute into gold the baser metals, the application of its principles, to the various arts connected with them, has certainly resulted in a golden reward. Did the occasion permit, it would be interesting to trace its connections with every art and science, where it has contributed to enhance the wealth, the comfort, and well being of individuals and nations; but it is proper that I should confine myself, at this time, principally to the consideration of it as connected with a single department, dental surgery. Chemistry has a most extensive application to general medical science.

It has furnished most important lights to guide the physiologist in his researches, and has solved many problems in this department, which but for its aid, would forever have remained inexplicable.

Modern chemistry has become the basis of materia medica; not only adding immensely to the former catalogue of remedies, but it has refined and rendered definite, both in composition and effect, those which hitherto composed it.

The medical jurist, by its aid, has been enabled in a thousand instances to decide whether crime or disease had been the agent in bringing death upon a fellow being; and where the former was reasonably suspected, or traced, it has often pointed out an effectual antidote.

But the kind and judicious counsels of chemical science, do not stop here.

Often when disease is racking the whole frame, when organ after organ is yielding under its influence, and when it would seem that the next moment all must bow under its sway, chemistry, by an analysis of some morbid secretion, may point to the organ most implicated, and hence to the remedial agent, which should be employed. Such are some of the applications of chemistry to general medical science. But to the *dental surgeon,* the aid of chemistry is still more available.

So far as medical science has contributed to the perfection of his specific branch, just so far is he indebted to chemistry as *its* auxiliary. Hence the dental surgeon is indirectly indebted to chemistry for all her donations to general medicine. But dental surgery is *directly* far more indebted to chemistry, than is any other department of the healing art; for notwithstanding as I have already stated, general medicine owes much to chemistry, for many of its perfections, yet it is nevertheless true, that conclusions based strictly upon the laws of affinity, as applied to highly vitalized parts, are often fallacious.

The laws of vitality are wholly opposed to those of chemical affinity, and the former doubtless always modify, and often wholly counteracts the latter. These two principles are at perpetual war with each other. While health remains, destructive affinity is apparently dormant, but no sooner are the powers of vitality weakened, than chemical affinity begins to tyrannize over her receding foe; and the instant the latter relinquishes the field, the former asserts her entire dominion ; and dissolves into its original elements, the citadel no longer protected by the force of vitality. By virtue of this principle, we find, for example, that the gastric juice, which readily dissolves the most firm animal tissues, when deprived of life, has no effect upon the stomach, when in a healthy state ; an example of the entire suspension of affinity, by the force of vitality.

If these observations are true, it must follow, that the physician, in order to calculate with any degree of certainty the results of remedies, as based on chemical laws, must constantly take into account the modifying influence of vitality.

But this is a field of investigation greatly deficient in correct data ; so much so, that many have been led to discard, altogether, all chemical theories, as applied to the living system.

But the application of chemistry to dental surgery, is wholly freed from all this ambiguity ; and this is the only department of the healing art, where the laws of affinity can be safely relied upon, and where they are not even modified by the living principle.

This view of the subject, is of course confined to the teeth themselves ; and mainly to their external covering, or the enamel ; which, with reference to chemical affinity, may be regarded strictly an inorganic substance. In all our observations upon the teeth, we should constantly keep in view the two different structures which compose them ; the bone and the enamel. The bony structure, composes much the greater portion of the tooth, and possesses vitality in a feeble degree—is endowed with blood vessels and nerves, and is doubtless to some slight extent, capable of resisting chemical affinity.

This structure is covered by a substance, differing from it very materially, both in composition and organization. The latter, which is the enamel, contains scarcely a trace of animal matter, and is neither endowed with blood vessels nor nerves, nor does it offer the slightest resistance to the action of chemical agents.

It is composed almost wholly of different salts of lime, and is designed as a protection to the bony structure. When this covering is destroyed by caries, it uniformly results in the destruction of the whole tooth; no portion of which is capable of self-restoration, as is every tissue possessed of any considerable degree of vitality.

After the simple statement of these few facts, it will not be hard to perceive the paramount importance of chemical knowledge, to the dental surgeon. We have on the one hand the teeth, with a definite chemical constitution, and on the other, an extensive list of articles liable to be brought in contact with them, whose chemical affinities are also unalterably fixed.

Now, to form a correct opinion, as to whether any of these, as applied to the teeth, will prove harmless or injurious, either directly, or by the fermentation which many of them undergo in the mouth, it must be evident that a most thorough knowledge of their relative affinities, is indispensable. It is now generally conceded, and it is a proposition clearly demonstrable, that what is termed caries in teeth, is nothing more or less than a chemical decomposition of the teeth; and when we reflect that there are many substances which are frequently brought in contact with them, which are capable of dissolving the enamel in a few hours, we need not wonder that their destruction is so common; I may almost say so universal.

The most destructive substances as a class, are acids, either directly applied, or generated from food which is often retained till fermentation takes place.

For example, I will notice the effect as shown by actual experiment, of a few articles which are familiar to all.

Acetic acid, or common vinegar, dissolves the enamel of a tooth when exposed to its action, at blood heat, in from forty-eight to sixty hours. Citric acid, or the juice of the lemon, produces the same effect in from thirty to forty hours, and moistened raisins, through the agency of the tartaric acid, which they always contain, in the form of cream of tartar, produces a similar result in less than twenty-four hours.* Did chemical knowledge enable us to account for, or prevent, only an occasional case of caries, the claim of this science upon the dentist would

* See American Journal of Dental Science, vol. 4, No. 1, article Dental Caries, by the author, pages 31 to 43, for a more detailed account of a series of experiments upon this subject.

be proportionately lessened; but when we reflect that caries in all cases, is the direct result of chemical affinity, of some one or more of the thousand articles, which are liable to be brought in contact with the teeth, can any one, making the slightest pretensions to skill in dental surgery, excuse himself for ignorance of chemistry?

Were it admissible farther to trespass upon your patience, I would pursue its connection with other departments of dentistry, but I shall leave this for a future occasion; simply adding that chemistry bears to dental surgery, the relation of a most faithful handmaid; ever needed, and ever ready to solve questions, which must present themselves at every turn, and in every department of your practice, and which without such aid, would ever remain inexplicable.

To the subjects thus far introduced, as claiming your particular attention, I have devoted much more time than can be bestowed upon the remaining branches of medical science; more perhaps than the occasion demands.

My only apology lies in their extent and paramount importance as elementary branches both of general medicine and dental surgery.

Not even an intelligible definition of disease can be given, without presupposing some knowledge of the *healthy structure* and *functions*, of the different organs, nor can the cause of their derangement be successfully traced, or remedial agents be safely and unerringly applied, without a practical knowledge of the laws of affinity.

But as important as are these elementary branches, they are of little avail, either to the physician or dentist, unless the principles to which I have referred, and the rules deducible therefrom, be applied to the study of disease, and the appropriate remedies.

This introduces two other branches of medical science, *pathology* and *therapeutics;* the former of which relates to the study of organs under disease, and the latter to the application of remedies. It is needless to observe that these two departments are inseparable. Nor need much time be spent in exhibiting their importance to the dental student, after having shown the anatomical and physiological relations, which the dental organs bear to the rest of the system. The study of anatomy and physiology would enable you to discover the existence of disease, by giving you a standard of health, both in structure and function, and the principles which these branches develope, may show you that diseased action might, and probably would be transferred from one organ to another, but neither would instruct you as to the nature of the disease in question, nor of the precise organ or organs which would suffer most by sympathy; nor yet of the remedies to be applied. These inquiries belong to pathology and therapeutics. They are subjects, with the general principle of which the dentist should be familiar; and especially with their application to his specific department. I have now enumerated those branches of medical science, most important to the dental surgeon, and have endeavored to set forth some of the many reasons why they should be faithfully investigated. But are not all these branches faithfully taught in our MEDICAL SCHOOLS? and if so, where is the necessity of dental colleges? If it be a fact that the elementary branches of medicine, are necessary to the well educated dentist, and that the kind of attention which these receive, at our medical schools, is well calculated to impart to the student in dentistry *practical instructions*, then have I proved too much for my purpose, and many of you have made unnecessary sacrifices to secure the advantages of a dental college, when a medical college, perhaps in your immediate vicinity, would have served you as well. This brings us back to a direct examination of the question, with which we started, viz. do dental colleges possess peculiar advantages over any other means of securing a dental education?

This is an important question—important to you and the public, whom you are preparing to serve, and on the decision of which, depends the prosperity, if not the continued existence of this and similar institutions.

In this utilitarian age, no institution can long exist, unless the wants of community demand it, and unless those wants are better served than at institutions of long standing.

Although I can look to one of our best medical colleges, as my *alma mater*, and although, hence, my prejudices have all been in this channel; and, while I regard the elementary branches of medical science indispensable to the dentist, yet, after a thorough examination of the subject, I am fully prepared to take the position, that the instruction given in medical colleges, though well adapted to the purpose for which it is given, is by no means calculated to impart to the dental student that informatton which he most needs, in order to insure him success in practice.

It would by no means necessarily follow, that because two sciences or arts were based upon the same elementary facts and principles, that the same course of instruction would be adapted to both. Nay, if their applications were different, it would follow that, to prepare students to practice each respectively, the course of instruction must be different.

It is but a few years since agriculture has been taught or practiced upon scientific principles; yet every principle upon which it depends, has been familiar to the *general chemist* for more than half a century.

Mr. Liebig, whose researches have shed such light upon organic chemistry, and who has wrought such great changes in agriculture, by no means discovered the principles upon which this new science is based. These had been taught by every professor, and learned by the student in general chemistry, for the last fifty years. Mr. Liebig's merit consists chiefly, if not wholly, in applying principles, already known, in a new way, or to new objects. But the result has been wonderful; and the fact that his discoveries have been confined to *new applications*, rather than new principles, detracts nothing from the honor due him.

Now, which of you, wishing to become a scientific farmer, would apply for instruction to the general chemist, who, perhaps, is ignorant of the specific applications of the science in which you were most interested, and whose applications were dissipated among the thousand arts and sciences, which are more or less dependent upon chemical principles.

It is easy to see the absurdity of such a course, were it possible to secure the instructions of those especially skilled in this department, and whose every application of the general principles, which are as perfectly taught as by the general chemist, is directed to the specific end you have in view. Much less would you apply to one for instruction, where every application of general principles was to be made in a channel calculated directly to *divert* your mind from the chief object of your pursuit. Again, were it your object to pursue chemistry as the basis of some of the arts, having no connection with agriculture, as the art of dying, or the improvement of steam power, or metallurgy, you would by no means place yourself under the tuition of Mr. Liebig, but, on the contrary, you would seek that course of instruction where the principles, *as they were presented*, would be applied, as far as possible, to the subject in which you were specially interested.

Again, were it your object to pursue mathematics, with a view to qualify yourself as an accountant, you would by no means seek, for this purpose, that school where mathematics were wholly applied to civil engineering; although each might be equally thorough in teaching the principles of the science. You have, doubtless, already anticipated me, in the application I am about to make of these observations. They clearly exhibit the impolicy, if not the absurdity, of pursuing any department of art or science, in those institutions where every application which is made of the principles taught, are directed to ends not consonant to the *direct* object of your pursuit. But let us make a more direct application of these observations to our subject. If we examine the different branches of medical science, as taught in our medical colleges, we shall perceive that quite as great a disparity exists between dental surgery and general medicine, in their practical applications, as there is between chemistry as a general science, and agricultural chemistry.

For example, the professor of anatomy exhibits the different parts, their structure, and relative position. But mark you; his applications are, almost without exception, to subjects in which the dental student is, to say the least, not directly interested. He exhibits one part as involving some great surgical operation; another, as being of peculiar interest in the treatment of this disease, and another of that. Sometimes he is imparting special instruction to the general pathologist, sometimes to the general practitioner, and again, to the general surgeon. In short, he applies his instruction to every branch, *except dental surgery.*

From the chair of physiology are taught, truly, the offices, and connection of different organs; but here too, the applications are widely variant from those which would most interest the dental surgeon. From the chair of chemistry are taught the general principles of the science, but to what are they applied? The student from this chair is made acquainted with many of the different articles used as remedies, and their mode of preparation. The professor, in this department, applies the general principles of his science to explain pathological phenomena, to pathology, and to show what substances are *incompatible with each other.* But from what professor of a medical college, has the dental student learned the chemical compatibility or incompatibility of the various articles used for food and condiments, or as medicines, with the *teeth?*

What man, occupying this position, has marked out and described a scientific plan, by which these useful organs might escape the ravages of protracted illness; and especially of the remedies employed to effect its cure?

Examine the array of subjects comprising the course given from the chair of *theory and practice*, and how many of them will you find coming under the head of dental subjects? Of the almost endless number of diseases here discussed, fortunate is it if those connected with the teeth receive a passing notice.

If you will examine the subjects discussed from the chair of general surgery, you will find the same conclusion will hold good. True, the discussion of any disease must often exhibit facts and principles, in which every student, of any branch of the healing art, is interested; but the mere *incidental* exhibition of principles, unconnected with direct application, is not what the dental student should seek, or be satisfied with, in the course of instruction which is to prepare him for the exercise of a responsible and difficult profession.

It has been the business of scientific dental surgeons, from the time of Hunter down to the present, to cull from general medical science, those facts and principles having a direct bearing upon this particular branch; and to arrange them into a new and specific science; and it is not difficult to perceive, that the dental student, who undertakes to glean from medical science, as taught at the present time in medical colleges, those facts and principles which are to constitute the basis of his practice, places himself back to a time when this was the only means from which to derive instruction.

Such are some of the objections to medical colleges, as furnishing proper instructions to the dental student, when dentistry is viewed as a science. But they are infinitely greater, when applied to this subject as an art.

In the practice of no art is there so great a demand for nice and extensive acquirements in mechanism, and indirectly upon the sciences upon which these different mechanic arts are founded. The various mechanical appurtenances which are used by the general surgeon, are either very simple, or are articles of commerce, found in the shop of every apothecary. If the surgeon is called upon to restore a fracture or dislocation, he employs an apparatus which has perhaps served every patient he has ever had. If he wishes to perform an operation, he has only to select the appropriate instrument, from a case containing every variety; the perfection of which has been the result of the combined ingenuity of a thousand geniuses, directed for a century to the improvement of this particular department.

Far different is it, in practical dentistry. In this department, the operator is often obliged, not only to manufacture his own apparatus, but actually to invent it for a given occasion. A mere description of almost any operation, connected with dentistry, would clearly demonstrate this position.

As an illustration, we will suppose the following case of regulating, one by no means uncommon: A child is presented, whose teeth are irregular among themselves; the circle of the upper jaw contracted, so as to be much smaller than that of the under jaw, and the latter protruding, so as to bring the front under teeth, when the mouth is closed, far forward of the corresponding ones of the upper jaw.

I need not say, that to remedy these several difficulties, requires a kind of skill not acquired or acquirable at a medical college. It is an easy matter to correct irregularities of the teeth, when only a portion of them are out of the proper circle. In this case you make a fulcrum of those in the right position, over which you can apply your levers, to bring the others in place; but where the entire circle is wrong, and especially where it is contracted, the difficulty is greatly enhanced.

The boast of Archimedes, that he would move the world, but give him a fulcrum on which to rest his lever, may be much more easily realized, than the moving of a single tooth, where there is no chance to apply to it the requisite power. But, in the case supposed, it is not only requisite to regulate the teeth among themselves, and to increase the whole circle of the upper jaw, but it is also as necessary that the position of the under jaw entire should be changed.

Now the mechanical appliances requisite to overcome this last difficulty, constitute a still greater demand for the exercise of skill and ingenuity. Turning our attention to the field of mechanical dentistry, we shall find this no less ample, in the scope it gives for the exercise of skill, contrivance and taste.

The dentist, in this department of his art, unlike the general surgeon, has to consult every mechanic art, and glean from them the skill without which he can make no advance; and, in the application of this skill to his peculiar art, unlike all other artisans, he is required to adjust and adapt his fabrics to living parts; and that, too, without impairing or endangering their health and safety. This remark applies particularly to the construction and insertion of artificial teeth. I might proceed to point out the particular difficulties which are involved in these operations; but the mere fact, that of all the artificial substitutes for natural teeth, which are prepared by dentists of every grade, not more than one case of ten meets fully either the expectations of the operator or patient; and, moreover, that in a very large portion of cases, these fixtures but prove a source of annoyance, laying the foundation for the destruction of other teeth, and involving, many times, the whole mouth in disease, is sufficient to show that there are difficulties connected with them, which are neither mastered nor even appreciated by a majority of operators. But, of all the various operations connected with the teeth, that of filling or plugging them is at once by far the most important and the most difficult.

If this operation be timely and successfully performed, it supersedes the necessity of all others. The operation of plugging a tooth is not only one nice and difficult in itself considered, but the difficulties are greatly enhanced, nay, multiplied almost ad infinitum, in consequence of its being performed within the cavity of the mouth.

You are, of course, aware that there is no conservative property, strictly so speaking, in any material used for filling teeth.

The end to be gained by this operation is simply to protect the portion of the tooth already affected by caries, from the farther action of those external agents which originated it, and by whose action it is progressing.

Now, taking, in connection with these considerations, the fact that these destructive agents are almost uniformly in a fluid state, you will readily see that a filling, to be efficient in arresting decay, must be so perfectly inserted, as to be wholly impervious to these fluids.

The mere fact that a plug remains in a cavity, is no guarantee that the tooth is safe.

If a person had a valuable casket of jewels, which was to be exposed to the action of an element that would deface or destroy them, with what scrutiny would he examine the envelope, and, if he should find it imperfect, so as to endanger, or perforated, so as to expose the valued treasure, with what solicitude would he seek the hand of skill and experience, to remedy the defect. Can such skill and experience then be dispensed with in filling the cavity of a tooth, where the operator has not only to attain the same end, but to do this under the most unfavorable circumstances; where extrinsic difficulties are to be contended with, which often, of themselves, are sufficient to thwart ordinary skill and perseverance?

This operation upon the teeth is to be performed in the cavity of the mouth, upon a living, sensitive tooth; often when the parieties of the cavity have become exceedingly frail, from the progress of the decay; not unfrequently upon the lateral surfaces of the teeth, where the space through which the cavity is to be reached, is necessarily so limited as greatly to curtail the efforts of the operator. These plugs in the teeth, as an essential quality, must be perfectly solidified throughout, making often a demand for the application of great force, to which there are many limiting circumstances. Again, they must be placed there perfectly dry, an indication, the fulfilment of which frequently constitutes one of the greatest difficulties the dentist has to contend with; as the salivary glands, by the irritation of of the operation, are excited, and an extraordinary amount of saliva is thrown into the mouth during the operation. Add to the difficulties above-mentioned, those growing out of tension of the muscles of the cheek, the occult situation of cavities, the great length of time during which all these counteracting obstacles must be controlled, to say nothing of the perplexities arising from poorly prepared foil, and you will have a faint picture of some of the difficulties in the road to fame, as an operator on the teeth.

If in this operation, also, we measure the difficulties by the failures which have so generally attended it, we shall find their magnitude by no means diminished; for, of all the operations which the dentist is called upon to perform, in none have failures been half so frequent. And that this want of success has arisen from its intrinsic difficulty, is sufficiently shown by the fact, that often the same person, who is entirely successful in almost every other dental operation, has utterly failed in this. Now, which of the difficulties, as presented in this description, is the student of a medical college instructed how to overcome? Nothing can be more absurd than to suppose that a degree from such a source, however well it may have been earned, will meet the exigency. Were I to enumerate every requisition for particular kinds of knowledge, which is made upon the practical dentist, and seek to meet them through the avenue of medical colleges, the effort would necessarily end in disappointment. But let us interrogate experience, and inquire whether physicians, although skilful as such, have shown themselves competent to discharge the duties of a dentist. So far as my own personal observation goes, I can confidently say, that they are generally, if not uniformly, unprepared to assume such duties; not even as safe dental advisers, much less as dental operators. During my attendance upon two full courses of medical lectures, the latter with the view to qualify myself to practice dentistry, I do not remember to have heard the subject of practical dentistry alluded to but twice. On one occasion, the professor of theory and practice of medicine, in alluding to the operation of filling the teeth, gave it as his firm conviction, that *tin foil* was as good a material for this purpose as gold! and that it would always be used, was not the dentist's *pocket* consulted!!

On the other occasion, a learned professor of surgery, in remarking upon the pathology of caries, attempted to show that the decay of the teeth, always originated from an inflammation of the bony structure, and that caries, hence, uniformly commenced internally; and of course was independent of external agents. Now, I need not say that both of these opinions, are wholly incompatible with correct practice. Do physicians generally, in treating chronic diseases, nervous affections, and the thousand ills consequent upon impaired digestion, as well as those affections originating in local irritation, look well to the condition of the mouth and teeth, to see whether they do not there find sufficient cause for the existing difficulty.

The inquiry of the physician, so far as regards the mouth, is generally confined to the tongue, as indicative of the state of the stomach, forgetting, or never having learned, that a healthy state of this organ is entirely incompatible with certain diseased conditions of the mouth.

Here the physician seeks for information through that avenue, to which his attention has been directed; and why should it not be so? For in what medical work, or by what professor has he been taught to do otherwise.

Again this great lack of knowledge, or at least a right application of it, is evinced by physicians, in their total neglect of the welfare of the mouth, during the illness of their patients. How often are the most beautiful sets of teeth entirely ruined, during even a short fit of illness. Nor is it the disease to which the physician is giving his attention, that is the active agent in this destruction, but it is his remedies. This is exemplified in the administration of the various acid tonics, gargles, &c., and in the general neglect of those means of care and cleanliness so important, especially during illness.

But I need not particularize; suffice it to say, that few are so fortunate as to recover from any thing like protracted illness, without their teeth being either ruined, or materially injured; a result which if it is not always attributable directly to bad management, is one which may always be avoided, by a judicious application of that knowledge, in the possession of every *well educated* dentist.

The impracticability of receiving that instruction which will impart this information, at our medical colleges, is pretty generally felt and acknowledged, and it has been proposed to supply the defect by adding a *dental chair*, to those now existing in these schools.

This proposition has been ably advocated, and it is certainly one, bearing upon its face, much plausibility. Such a chair as an appendage to a medical college, must have for its aim, the accomplishment of one of the three following objects, viz. Either to instruct the medical practitioner as such, or to enable the physician to append practical dentistry to his cardinal profession; or to qualify students to practice dental surgery alone.

If the first of these three objects, were the only one had in view, I would most cordially approve of the project; for it is no less important to the physician than to the dentist that he should thoroughly understand the pathology and morbid effects of the diseases of the dental apparatus, as well as the effects resulting to it from disease in other parts of the body.

Nay more. He should be familiar with all the elements necessary to good dental *operations*, that he may be thus enabled to co-operate with the worthy portion of the dental profession in enlightening the public, and in the suppression of dental quackery.

But the establishment of such a chair, either with the view of amalgamating dental and medical *practice*, or of qualifying students for practising dental surgery, as an independent profession, is a proposition, the feasibility of which, admits of serious question.

It is urged by the advocates of such a chair, that it would enable the young physician to make a handsome saving from *dental* practice, by spending in this way his leisure, of which he is too apt to have an abundance. But why has the young physician, fresh from the seat of medical science, so much leisure? Is it simply because he is young in years, or is it not rather because the community at large, well understand that the young graduate, in order to become a skilful practitioner of medicine, must yet devote much time to reading and observation, which he is now just prepared to do profitably.

Shall his leisure, then, be devoted to the practice of a profession, entirely unlike his primary calling? Can he take medicine in the one hand and dentistry in the other, and carry both along, even with profit to himself?

This certainly would be an attempt to serve two masters, very dissimilar in the requisitions which they respectively make.

This attempt where it has been made, has generally resulted in the abandonment of one or the other, and where this coalition has continued, I have never been so fortunate as to find the man, who did justice to, much less excelled, in either.

It is, indeed, contended by many, and I may perhaps say by a majority of our most eminent dentists, that the two departments of *dentistry*—the surgical and mechanical should always be assigned to different hands; and that this is necessary to the attainment of great excellence in either. But if excellence in each of the departments of dental surgery, is with difficulty attained by the same individual, much less can this profession, as a whole, be regarded in the light of a mere appendage; and its different departments successfully cultivated and practiced, in connection with the profession of medicine; which of itself is a subject, when confined to its legitimate limits, too extensive, to be fully comprehended and successfully practiced, without an expenditure of more time and money, than can generally be devoted in this country, to the acquirement of a profession.

Whether, then, we view these two professions with reference to the magnitude of each, or with reference to their dissimilarity, we shall see that their *practice*, cannot be well combined in the same individual.

The same considerations, as plainly declare, that *the interest*

*of each profession*, demand the separation. I have thus far gone upon the supposition that this superadded dental chair, was capable, of qualifying a student for dental practice.

But this is a supposition which admits of strong doubts, if it is not wholly groundless.

I shall farther attempt to show, that the *dental student*, whose mind is wholly directed to the acquirement of dental knowledge, would be inadequately instructed by such a course; and if this be so, much less would the medical student, whose mind was mainly directed in another channel, become qualified properly to discharge the duties of the practical dentist. I have already remarked that the course of instruction given by each of the professors, in our medical colleges, was prepared for and adapted to a specific end; one widely differing, in almost every practical detail, from dental surgery; and that every application of facts and principles which is made, is equally foreign to the object which the dental student has in view. Such a course might be profitable to the *experienced dentist*, who has already proved the great utility of these facts and principles as applied to his own profession, and who has had frequent occasion to regret his deficiency in them; but far better would it be for the *inexperienced student*, were only facts and principles exhibited. The different lectures, which were perhaps written out by the several professors in their closets at home, may be justly compared to so many different suits of clothes, cut for, and adapted to a given number of individuals.

Now, the dental professor of a medical college would find himself in the attitude of one, whose business it was, to select or manufacture from these ready-made clothes, garments for another class of individuals, differing materially both in stature and proportion; a task at once both embarrassing and laborious.

It might, indeed, be honorable for a dentist to wear a coat made from the cloak of a medical man, but in behalf of the faculty of this college, I would say, give us the *cloth* before it is made up, and we will endeavor to give you a more perfect fit, and at a much more economical rate. A chair of dental surgery, as an appendage to a medical college, must be a practical one; and the professor must pre-suppose the students already versed, in all the elementary branches, which this subject involves; together with their application; for *he* certainly could not make the requisite applications, which should have been made by each, without *absolutely repeating* at least the substances of their lectures; thus virtually assuming the work of the whole faculty. Such an one would be entitled to have inscribed upon his insignia, "E Pluribus Unum." But before being long engaged in this arduous undertaking, he would doubtless stop to inquire, how much this labor could be curtailed, compatibly with the interest of his pupils; and especially would he be inclined to do this, when he found that after all he could glean, and turn to good account, from the whole course, it becomes necessary to teach a host of new facts, and new principles, in order to do justice to his own subject. In pursuance of this inquiry, it would doubtless occur to him, that the subjects of several of his colleagues, might be wholly dispensed with; and that the useful material might be arranged under a more convenient head; and for the lack of these, he would supply others, more pertinent to the object of his own course. In short there can be but little doubt, that the experiment would convince him, that the whole course should be remodeled:—selecting those portions of medical science, particularly applicable to dentistry, adding what would still be wanting, and arranging the whole under a suitable number of heads; and assigning them to teachers, who, though engaged in different departments of the subject, should mutually direct their energies to the same end—to perfect the student in all that can pertain to dental surgery.

In other words he would doubtless become convinced, that it would be good policy, to transform his medical into a dental college. If he was not driven to this conclusion from the considerations already named, he would doubtless be, by the utter lack of time which could be devoted to this dental chair by students while attending medical lectures. In every respectable medical school of this country there is from five to eight chairs, and the student is called upon to attend this number of lectures daily. Add to this, the time required for examinations, dissections, witnessing surgical operations, and for incidental matters, and who cannot see, that little would be left for special attention to the department of dental surgery. It must be evident to every reflecting mind that it would be vastly too limited to receive instruction even in the *theory* of dentistry. But a proper course of dental instruction should by no means] be confined to theory. Were this admissible, the student might well save himself the trouble and expense of attending lectures at all; for mere theory can as well be learned from books as from a teacher. In this institution each student is required to be engaged in actual practices from four to six hours each day, beside seeing all the different operations performed time and again by the different teachers.

The supposition that *theory* alone will qualify students as practitioners of dental surgery is as absurd as to suppose that Sivori, or Ole Bull, had attained their almost superhuman skill upon the violin by merely *listening* to the enchanting strains of the immortal Paganini. To acquire that dexterity of hand necessary to perform the nice and delicate operations upon the teeth, as in the execution of music, the *hand* must be educated, or failure is inevitable.

That dental colleges may be so constituted, as to afford to the dental student, advantages pre-eminent above any other means of instruction, there can be no doubt.

I have endeavored to point out some of the difficulties connected with receiving proper and full instruction at medical colleges, even on the supposition that a dental chair be appended to them; and, it hardly need be observed, that *private* instruction is seldom, or never what it should be, particularly in the *science* of dentistry, when we reflect that those most capable of imparting it, have always the least time to devote to teaching.

Indeed, with an occasional exception, you will find that our first class operators do not receive students into their offices.

They too well know the difficulties connected with obtaining a thorough dental education, and are hence unwilling to assume the responsibility of such instruction; while it is common for those who are ignorant of even the first rudiments, to "send out from ten to twenty-five per annum."

If such be the claims of dental colleges, why deny them an independent existence?

Such a denial can only be predicated on the supposition that the subject is too trivial to be regarded, or taught, other than as an appendage, to one more commanding in its claims.

But is dental surgery so limited in her requisitions, or her capabilities; or so doubtful in her claims, that she must needs attach herself to some well grounded science, to command respect?

Let her splendid achievements, which can be attested by thousands, answer the question. Again, is the demand for her aid so limited, as to countenance the position, that no exertion need be made to cultivate this branch of the healing art?

Let the whole human family, who have suffered more or less from dental diseases reply. It may indeed be safely assumed, that the aid of no profession is so universally implored, as that of dental surgery. Nor have the resources of any profession proved more nearly commensurate with this demand.

But to command, fully, every resource which a high cultivation of the science and art of dentistry affords, a more intimate and extensive acquaintance with every collateral science and art is requisite, than in the prosecution of any other calling.

There is no science connected with material substances, which may not be profitably consulted by the dental surgeon; nor art which may not directly or indirectly lend him aid. But, unfortunately, the full measure of these resources, is limited comparatively to a few; and this must ever be the case, while the *means of acquiring* a correct, thorough and systematic dental education, are as limited as they have hitherto been.

Till the establishment of the Baltimore College of Dental Surgery, 1839, no institution existed, expressly for the purpose of giving instruction in this department.

Up to that time instruction was exclusively confined to private tuition; a policy clearly based upon the false supposition, that dentistry was a subject of small magnitude. For some time, the scheme of establishing a college expressly for this purpose, was regarded by the community, and even by many of the profession, as chimerical. But thanks to the untiring exertions of its enterprising founder, the enlightened policy of the state legislature, and to the cheering countenance and liberal support of the citizens of Baltimore, the experiment has been fully and successfully tried; the practicability, and the great public utility, of such an organization, has been fully proved; and the objections arising from the fears of friends, or the ill-boding prognostications of enemies, have been most triumphantly refuted.

This experiment has not only been successful in itself considered; but it has become the corner-stone of a new, and more enlightened policy; both in regard to the public and the profession.

Let us see to it that we lose not this vantage ground. If we do this by making our facilities for imparting instruction fully commensurate with the demand, by making our diploma exclusively the reward of merit, it will require no enthusiasm to induce a strong confidence that the students sent abroad from this institution, will practice their profession, with honor and profit to themselves, with benefit to their patrons, with credit to us; and that they will prove efficient, living witnesses of the feasibility and great public utility of dental colleges. In proportion as such practitioners become the occupants of the various fields, will empiricism and imposture be supplanted by science—bombastic pretensions by modest merit—and public suspicion by a just confidence in the meliorating powers of science—the blind avidity for secret and patented nostrums, by a just respect for, and an intelligent appreciation of those resources, of science and art, which in the hand of the enlightened and honest professional man, are the only legitimate agents, for meliorating the sufferings incident to humanity. May the time soon arrive when men deeply imbued with the love of science, and skilled in its application to dental surgery, may be so thickly scattered throughout the length and breadth of our land as that their mutual and combined light shall leave no spot of darkness to shield from full public recognition the devices of the dental empiric.

